# Palliative and End-of-Life Care Service Models: To What Extent Are Consumer Perspectives Considered?

**DOI:** 10.3390/healthcare9101286

**Published:** 2021-09-28

**Authors:** Bruce Rumbold, Samar M. Aoun

**Affiliations:** 1 Public Health Palliative Care Unit, School of Psychology and Public Health, La Trobe University, Melbourne, VIC 3086, Australia; 2 Perron Institute for Neurological and Translational Science, Nedlands, WA 6009, Australia

**Keywords:** palliative care, end-of-life care, consumer preferences, models of care, hospice, hospital, residential aged care, home care, public health approach, compassionate communities

## Abstract

This article presents evidence found in a search of national and international literature for patient preferences concerning settings in which to receive palliative care and the appropriateness of different models of palliative care. The purpose was to inform end-of-life care policy and service development of the Western Australian Department of Health through a rapid review of the literature. It was found that consumer experience of palliative care is investigated poorly, and consumer contribution to service and policy design is limited and selective. Most patients experience a mix of settings during their illness, and evidence found by the review has more to do with qualities and values that will contribute to good end-of-life care in any location. Models of care do not make systematic use of the consumer data that are available to them, although an increasingly common theme is the need for integration of the various sources of care supporting dying people. It is equally clear that most integration models limit their attention to end-of-life care provided by health services. Transitions between settings merit further attention. We argue that models of care should take account of consumer experience not by incorporating generalised evidence but by co-creating services with local communities using a public health approach.

## 1. Introduction

Over the past decade, the claim that “end-of-life care is everyone’s responsibility” has appeared in national and regional palliative care policies around the world. Comprehensive strategies through which this shared responsibility might be discharged are, however, far less common. In most cases, it is translated as a desire for ‘community involvement’ implemented through consumer consultation during policy and service development. This consultation is often individualised by involving ‘representative’ consumers, with little information provided as to how representative these selected consumers might be. However, more jurisdictions are beginning to draw upon a growing amount of quality assurance work on consumer experience in their policy formation.

In response to Recommendation Nine of the Western Australian (WA) Parliament Joint Select Committee on End-of-life Choices [1], the WA Department of Health commissioned an independent review of consumer perspectives of palliative care service models. The independent review comprised three phases: a literature review, a cross-sectional consumer survey and consultation forums with service providers.

The Department intended this literature review to clarify the extent to which end-of-life services address consumer needs or incorporate consumer feedback. The literature review is based on the global English language literature, but Australian studies are selected to illustrate findings where they are available.

## 2. Objectives

The objectives of the whole independent review, including this literature review, were to:

1. Review consumer preferences on receiving palliative care (at home; residential care; hospital; hospice; mix).
2. Describe and investigate existing models of palliative care and whether the palliative care needs of consumers are met by each model.
3. Review the appropriateness of each service model with respect to meeting the needs of consumers and care providers.

## 3. Methodology

Given the timeframe allocated for the review, it was necessary to develop a rapid review methodology [2]. While the debate continues around the definition of a rapid review [3], they typically include ‘shortcuts’ that circumvent some of the more time-consuming aspects of systematic or even scoping reviews. Rigour is to some extent sacrificed for timeliness, and the quality of the review depends upon the shortcuts taken.

Our interest here was in identifying, as far as possible, a working consensus on evidence that would inform the 3 objectives indicated above. We decided that we would focus first upon systematic reviews and that we would use a Google Scholar search to identify these. The choice of Google Scholar was based both upon this database’s capacity to identify relevant, high-quality literature [4,5] and its inclusion of some grey literature. In searching for data to inform a policy review, we were interested not only in peer-reviewed literature but in data sources taken into account in other policy formation processes. The material identified in a Google Scholar search would then be checked and complemented by further targeted keyword searches of databases, principally CINAHL and PubMed [6], while further systematic reviews would be sought in the Cochrane Library and the PROSPERO International Register.

The initial Google Scholar search used keywords [systematic review] + [palliative care] + [consumer] and was limited to the previous 5 years, identifying 15,900 references. The time span of 5 years was selected because consumer perspectives have only been included routinely in policies over the past 10–15 years, and we thought it reasonable to expect that systematic reviews conducted in the past 5 years would include the literature on which these policies were based. It was hoped that a focus upon systematic reviews would enhance rigour and incorporate, through their review processes, a body of evidence prior to the search cut-off date of 2016. The search was conducted on 2 computers to check for algorithmic bias, but no significant discrepancies were found. The references were ordered according to relevance, and the first 1000 were hand-searched to identify material directly relevant to the study objectives. After the first 1000 it was clear that references were of decreasing relevance, relating principally to only 1 of the combined search terms.

To supplement this material, we searched for studies other than systematic reviews that connected consumer experiences with their settings. These searches were not limited to the previous 5 years, but our interest was principally in studies that reflected the experience of contemporary care systems. These searches in CINAHL and PubMed used keyword combinations including the following:

- [models] + [palliative care] + [delivery]
- [integrated] + [palliative care] OR [end-of-life care]
- [quality] + [evaluation] + [palliative care] OR [end-of-life care]
- [end-of-life] + [settings − home, hospice, hospital]
- [patient] + [family] + [informal] + [consumer] + [care *]
- [end-of-life] + [priorities] + [needs] + [preferences]
- [communication] + [information] + [decision-making]
- [patient] + [carer] + [reported outcomes]

Our interest here was developing insight into processes and experiences that lie behind the aggregated evidence reported in the systematic reviews.

## 4. Findings

The findings of the rapid review were presented in narrative form and organised according to the three objectives. A scan of articles found in the Google Scholar search showed that systematic reviews of patient and carer experiences at end-of-life do not always focus on the context in which care was provided, while systematic reviews of service models do not always attend to patient and caregiver experiences. We decided to report on these strands separately, as limiting our findings to reviews that explicitly link experiences with particular settings would have neglected a considerable amount of rich data. The references provided in this article illustrate key points of our findings: it is not possible to provide here a complete list of articles.

### 4.1. Consumers’ Needs and Preferences at End-of-Life

Consumers of end-of-life services potentially comprise the entire population, but population-wide surveys tend to be limited to broad issues such as a preference to die at home [7] or the desirability of assisted dying legislation [8]. Studying the preferences of actual consumers of end-of-life services selects a sub-set of the population who have chosen to engage with these services. The extent to which these findings represent the whole population of dying people and carers is unclear.

Studies often investigate preferences in terms of location of care or model of care, but findings are predominantly about quality of care. The location of care and model of care may be secondary to these qualities being realized, although, of course, quality concerns may inform a preference for site and style of care.

There is reasonable consensus about the experiences and responsibilities of different participants in end-of-life care. Concerns common to patients, family, physicians and other caregivers included pain and symptom management, preparation for death, decisions about treatment management, achieving a sense of completion and being treated as a whole person. Important to patients, but less to physicians, include being mentally aware, having funeral arrangements made, not being a burden, helping others and achieving peace [9]. Within each group, differing perspectives were found concerning decision-making about life-sustaining treatments, dying at home and talking about the meaning of death.

These differences can impact the quality of care. Misalignment between the medical system and patient/family values and priorities frequently contributes to consumer dissatisfaction with care [10]. Yet, even when goals of care are aligned, information sharing can remain a problem [11], in part as a result of the increasingly varied sources of information accessed by patients and family caregivers [12].

Caregivers’ effectiveness in responding to needs depended upon them knowing and adhering to patients’ wishes. The extent to which health services are responsible for supporting caregivers continues to be debated, but traditionally palliative care has regarded patient and family as the unit of care. Hudson et al [13], for example, reported success with a psycho-educational program that provides information and skills to support informal caregiving in the chronic illness aspects of palliative care. A recent Cochrane review [14], however, found mixed evidence for the value of psychosocial interventions across the illness trajectory. Candy et al. [15] noted some evidence that strategies to help caregivers indirectly via patient care seemed to have some effect. Most of the other interventions that attempted to work directly with caregivers did not include practical support, although support in acquiring practical nursing skills has been shown to be important to caregivers [16]. Aoun et al. [17] used a stepped wedge cluster trial (n = 620) of a carer support intervention in community palliative care in WA, finding that priority support needs identified by caregivers included knowing what to expect in the future, having time for yourself in the day and dealing with your feelings and worries. Their intervention reduced caregiver strain before bereavement and had positive outcomes post bereavement by achieving the preferred place of death through an agreement between patients and their caregivers [18]. The authors concluded that, within palliative care, the intervention with the potential to have the greatest ‘reach’, and available to a wide population of carers, is the one that adopts a comprehensive, person-centred approach to carer assessment in routine practice, ensuring that carers have the opportunity to consider and express their support needs so that service providers can deliver support tailored to their individual circumstances.

A theme emerging in recent studies is the need for practitioners to give more attention to patients’ experience of living with their illness, not just to providing information about the diagnosis, prognosis, treatment and symptom control, important though these remain. Wang et al. [19] identified unmet care needs as emotional support, fatigue and being informed about benefits/side effects of treatment. Sheehan et al. [20] showed how treatment burden for patients and families includes understanding the condition, juggling monitoring and adjusting treatments, engaging others for support, time and financial issues. The burden does not end with a patient’s admission to hospital, hospice, or aged care [21] but transforms into a different set of responsibilities, relationships with professional caregivers, care decisions and discharge planning.

Linking illness experience with other aspects of daily living is vital for preserving quality of life. For example, in a small NSW study of older adults with terminal illness [22], participants describe their needs in the following domains: quality of life, sense of control, life on hold, need for health system support, being at home, talking about death and competent and caring health professionals. Rand et al. [23] found that patients with advanced cancer had life goals that resembled those of healthy populations and that treatment goals were separate from these life goals. A capacity to align treatment and life goals shaped the final stages of illness, and those who valued cure the highest had the worst psychological adjustment [23]. This supports the case that early palliative care intervention, introduced alongside treatment options allows better preparation and facilitates informed choice [24,25]. Collins et al. [26], however, reported that consumers’ perceptions can work against early introduction of palliative care if palliative care is associated in their minds with diminished care, diminished possibility and diminished choice. Aoun [27] proposed early integration of a palliative approach in the care plans of people diagnosed with Motor Neurone Disease (MND), arguing that this can optimise their quality of life by relieving symptoms, providing emotional, psychological and spiritual support pre-bereavement, minimising barriers to a good death and supporting the family post-bereavement. Knowledge and expertise need to be extended beyond the domain of specialist palliative care services to include the full scope of health and community-based services providing care, mostly at home.

Dalal and Bruera [28] were more positive about the contribution of early palliative care intervention, in part because of the effect of palliative care in reducing costs of (futile) aggressive treatment and thus reducing financial pressure on US families. Hospice participation in care through attention to values and multidimensional needs allows care to be planned and reduces costs associated with a narrow focus on the disease, which can cause further morbidity due to harmful interventions [24]. While these studies reflect the US context, financial constraints are also found to affect treatment choices and quality of life of cancer patients in Australia’s universal healthcare system [29].

Recent reviews point out that studies of needs seldom consider the full treatment pathway/cancer journey [30,31]. With advanced cancer increasingly experienced as a chronic illness [32], needs remain diverse, requiring greater attention to the illness experience of particular people. Other studies report similarly on the end-of-life needs of populations belonging to specific illness groups. Stow et al. [33] found the needs of frail people to be similar to those with advanced cancer, but that a frail person’s desire for reduced interventions was not always observed. In cases where palliative care was provided to frail people, their relatives often rated it lower in comparison to the relatives of those with cancer, presumably because palliative care staff lacked familiarity with frailty and the less predictable trajectory of dying. The implication here, that end-of-life care needs to be provided by people familiar with the particular needs of a population, is reinforced for MND [27,34] and intellectual disability [35].

#### 4.1.1. Collecting Data on Consumer Engagement

Most palliative care programs collect some form of internal consumer data, but few publish their findings. Consumer satisfaction scores are often collected by palliative care peak bodies, but this activity tends to be more a marketing measure than a focused enquiry into quality. To date, the only program that has consistently collected consumer data in granular detail across a health system is the Views Of Informal Caregivers Evaluation of Services (VOICES), commissioned by the UK Department of Health [36], and badged as the National Survey of Bereaved People (VOICES) once managed by the Office for National Statistics (ons.gov.uk). The survey investigated the quality of care delivered in the last three months of life for adults who died in England, using a sample of approximately 30% of all deaths over a four-month period selected from the death registration database.

The dataset continues to be used retrospectively [37] in various ways: identifying associations between the place of death and Advance Care Plans [38] or end-of-life experiences for particular populations such as people with intellectual disability [37]. Modified versions of the survey have also been used in comparing end-of-life care across specific settings [39].

The closest equivalent in Australia is the FAMCARE-2 tool (Family Satisfaction with Palliative Care), which measures satisfaction across four domains, management of physical symptoms and comfort, provision of information, family support and patient psychological care. The validation study [40] indicated lower levels of satisfaction in response to the subscales ‘provision of information’ and ‘family support,’ consistent with VOICES findings. FAMCARE-2 is administered periodically in selected services by Australia’s Palliative Care Outcomes Collaboration (PCOC). A survey of 1592 caregivers across 49 palliative care services in 2016 [41] found generally high levels of satisfaction and positive experiences of care. Scores were higher for inpatient care on three of the four domains, provision of information being the exception. Dissatisfaction with information provision was higher for older carers, particularly around carer payments, while home carers reported that information to support them with practical caring tasks was inadequate. These findings were consistent with the Australian EOL and Bereavement Study [42].

#### 4.1.2. End-of-Life Needs in Particular Settings

A few studies directly investigated patients’ perspectives of differing end-of-life services—home care, residential care, hospital, hospice—but it is still necessary to understand what each of these services provides and how they are aligned within that particular health system in order to compare the findings of the different studies or translate them to another context. Possible confounding issues are distinguishing if a hospital setting includes the care of palliative care consult teams or if residential aged care setting accesses care from community-based palliative care programs.

##### Hospital

Six themes common to patients and families – expert care, effective communication and shared decision-making, respectful and compassionate care, adequate environment for care, family involvement and financial affairs – were identified by Virdun et al. [43] as important in hospital end-of-life care. Patients added the theme of maintenance of sense of self, while families added patient safety, preparation for death, care for family after death and enabling patient choice at end-of-life. A recent study [44] in five NSW hospitals confirmed these domains and suggested two more, nutritional needs and access to clinical specialists. These findings were supported by a recent review by Wong et al. [45].

Seldom, if ever, were all these areas of importance adequately addressed. While not coinciding with the eight essential elements of Australia’s National Safety and Quality Health Service Standards [46] for comprehensive care at the end-of-life, the themes identified by Virdun et al. [43] were consistent with them. Bloomer et al. [47], in auditing hospitals against these national ACSQHC guidelines, found clear scope for improvement, particularly in patient-centred care, family involvement, describing and enacting goals of care and using triggers to prompt care. These are, in fact, all the essential elements for the process of end-of-life care [46] and presumably point to deficits in the underlying organisational pre-requisites. It might also be noted that the standards specify access to specialist palliative care advice as one of the requirements of any comprehensive end-of-life care plan.

Will et al. [48] conducted a review that underlined the importance of multi-disciplinary team care in patient satisfaction with care, particularly when teams comprise more than two professions (that is, include allied health practitioners) and engage the patient through more comprehensive team practice (that is, practice that involves more than a multidisciplinary ward round).

A recurring issue with hospital end-of-life care is non-beneficial treatment. Cardona-Morrell et al. [49] reviewed the treatment of older patients near end-of-life and found that on average 1/3 received non-beneficial treatments including dialysis, radiotherapy, transfusions and life support treatment and that up to 2/3 of admissions were not mandated by therapeutic need but a lack of alternative strategies for care. Taylor et al. [2], in a scoping review of initiatives to reduce inappropriate or non-beneficial hospital admissions, found a wide range of strategies being employed, but that evidence for their effectiveness was generally limited or absent.

Singotani et al. [50] pointed out that most studies of unplanned readmissions were evaluated from the hospital’s perspective in terms of patients’ failure in self-management or problems with integrated care. Studies differed as to which causes were preventable, nor did they take into account causes beyond the scope of the hospital. Trankle’s [51] small survey of specialist physicians even questioned whether a good death was possible in Australian critical and acute care settings, noting that 70% of all deaths were in institutions, and that 15–20% of those took place in ICU where a quarter of patients were ventilated and almost 40% died in pain.

##### Residential Aged Care

A Cochrane review found little evidence for the effectiveness of interventions intended to improve palliative care in nursing homes [52]. Tilden et al. [53] noted the problem of high staff turnover and the high personal and economic cost that works against quality of care. They argued that investment in staff training to improve knowledge and skills and increase job satisfaction would improve staff retention and reduce transfers to hospital for conditions—including end-of-life care—that should be managed on-site. There was some indication within the New Zealand context [54] that the nature and quality of end-of-life care can be contingent upon a General Practitioner’s engagement with a residential aged care facility. This finding is confirmed in studies of rural RACFs in the Monaro region of Australia, where monthly Needs Rounds involving RAC staff and local GPs increased capacity to provide end-of-life care [55].

Milte et al. [56] surveyed 17 nursing homes across four Australian states to ascertain the characteristics most valued by residents and family members. While residents receiving palliative care were excluded, these values have important implications for understanding the context with which palliative care ideally might articulate. Belonging (feeling at home) is of primary importance to residents, as is flexibility in the care routines provided by staff.

##### Residential Hospice

There are surprisingly few systematic reviews of the quality of residential hospice care. Weerakkody et al. [57], in a study involving 100 bereaved caregivers of people who died in a Toronto (Canada) hospice, found that quality (measured by the Quality of Dying and Death- QODD) was average to above average and that higher scores were reported by those whose relative had been admitted for more than one week. A possible explanation can be inferred from the evaluation by Lucey et al. [58] of patients admitted to Milford Hospice, Limerick, Eire. They found nearly half were unstable on admission, and it took three days for 70% of these patients to be stabilised. That is, longer admissions were needed to be of clinical benefit, let alone to access other forms of support provided in the hospice.

##### Home

Reviews by Stajduhar et al. [59] and Funk et al. [60] highlighted the need for a systematic study of home-based family caregiving at the end-of-life. Their findings identified the ways caregiver experience can be disrupted as demands change and different services become involved, outlined forms of support required, discussed barriers to receiving that support (often the reluctance of caregivers to articulate their own needs) and explored caregivers’ role in decision-making. A further theme identified was the rewards of caregiving as a source of comfort, strength and meaning alongside the stress and challenges involved. A Cochrane review found that home palliative care more than doubles the chances of dying at home and reduced symptom burden without increasing grief for family caregivers after the patient died [61]. Another Cochrane review of the impact of hospital at home programs (Shepperd et al. [62]) made similar findings.

A small Dutch study [63] identified medical proficiency in the primary care team, including clarity about procedures and pro-active approach, as essential elements of high-quality palliative care at home. Barriers to dying at home identified by O’Brien and Jack [64] were, from a service perspective, poor discharge planning, difficulty in accessing equipment and services and inadequate out-of-hours service provision.

Debate continues as to whether death at home should be an outcome measure [65]. While well-being may be greater at home, preference alone is not enough to ensure death at home and instead is influenced by other factors such as extended family support, availability of home care and affluence [66]. People who have had a stroke or people with dementia are less likely to die in their preferred place [67]. A longitudinal study to elicit the end-of-life preferences of terminally ill people who live alone was undertaken in community palliative care in WA [68]. Congruence between the preferred and actual place of death shifted from 53% based on preferences at baseline to 41% based on preferences at follow-up. For nearly half of the patients in this study, home was not their preferred location for dying. The authors suggested that the ability to die in the place of choice (rather than home) needs to be looked at as a possible indicator of meeting patient needs or as a quality measure in end-of-life care.

The critical role of the GP in supporting care in the home through linkage with specialist palliative care services is shown in the review by Carmont et al. [69]. A nationwide integrated information network for primary end-of-life care in Australia was designed and piloted, and early findings show that palliative care training for GPs improves the uptake of ACP in general practice [70].

#### 4.1.3. Comparative Studies of Care in Different Settings

The English National Bereavement Survey [71] reviewed care in the last three months of life and consistently found the overall quality of care in hospitals to be worse than any other setting, with 30% rating hospital care as fair or poor. Care in care homes, hospices or own homes was rated highly (80% as outstanding, excellent, or good). The overall quality of care ratings did not vary significantly for different illnesses, except that those responding on behalf of cancer patients gave more outstanding ratings (nearly 50%) than for cardiovascular patients (less than 40%). In 2015, but not 2014, females were perceived by respondents as receiving better care than males. Excellent ratings for quality of care were highest for hospices (76%) and lowest for urgent care services (26%). The majority of informal caregivers agreed that their information and communication needs were met. About 20% said that decisions were made about the patient’s care that the patient would not have wanted. An overwhelming majority felt the person had died in the right place, even if at times that place had not been the person’s expressed preference.

Sandsdalen et al. [72] conducted a study using the Quality from the Patients’ Perspective for Palliative Care (QPP-PC) measure of 191 Norwegian palliative patients in hospice inpatient care, hospice day care, palliative care units in nursing homes and home care. Perceptions of care quality were higher for hospice inpatient care than other settings, although perceptions of subjective importance did not vary across settings. In all settings, the prime area for improvement related to receiving information.

The Australian End-of-life and Bereavement Survey found significant differences in the quality of palliative care received between the three settings: community, inpatient/hospice and nursing home. More respondents rated inpatient settings as excellent/good (93%) than care in the community (81%) or care in nursing homes (73%) [42]. This, to some degree, contrasts with de Boer et al. (2017), who found relatives more positive about care received from palliative care teams at home or in hospice than in the hospital setting. Their study drew upon data from 14 European countries for deaths from a wide range of causes. They noted that the psychosocial support provided in the hospital or aged care was less than at the home or hospice and that the level of satisfaction with a setting also varied according to the illness from which a person had died. In the Australian study, most in the palliative care group had died from cancer, and the higher rating for inpatient care may reflect both the complexity of managing symptoms in this group and the wide availability of hospital palliative care consult teams.

This review of the evidence related to consumer preferences suggests that best practice is defined more by the qualities and values embedded in the care provided, not a particular program structure or setting. The most appropriate model of care will be one that is able to respond flexibly to this variety of needs across the illness journey, with that flexibility extending to end-of-life experiences.

### 4.2. Models of Care

Many health systems have over the past decade reviewed and revised their end-of-life care strategies, usually in an attempt to better integrate palliative care with the overall health system. The majority of these revised end-of-life service models are based on practitioners’ perceptions of patients’ needs. That is, although patients’ responses may be considered in revising services and in evaluating the effectiveness of the revisions, service design has tended to give priority to solving health service problems more than patients’ perspectives of their need of preference for care. A rich variety of reports and reviews is available from selected health services and peak bodies within their jurisdiction. Peer-reviewed studies more often focus on a single aspect of the multi-faceted end-of-life care system. Models draw upon such studies in the hope that the sum will be greater than the parts.

A theme common to the recent literature on models of care is the need for integration. It is equally clear that most integration models being put forward limit their attention to end-of-life care provided by health services.

#### Integrated Palliative Care

Luckett et al. [73], in a rapid review, concluded that effective palliative care models integrated specialist expertise with primary and community care services and enabled transitions across settings, including residential aged care. Their focus was on elements relevant to the Australian health system—that is, the terminology was aligned with Australian definitions. They also noted that:

- The use of volunteers has potential when other informal caregivers are lacking, but that this raises governance issues.
- GPs lack capacity due to workload and inadequate remuneration.
- Effective models such as the Gold Standards Framework [74] are not readily transferable to Australia.
- Case management is important, but also problematic given the multiple jurisdictions and care settings in Australia.
- Population-based models of palliative care should support case management via integration of Specialist Palliative Care (SPC) with primary and community care services. That is, they advocate a model of care that provides continuity through the illness course.

Reviews of palliative care integration in Europe have also been carried out [75,76]. The definition of integrated palliative care (IPC) developed by van Beek et al [76] is:

“Integrated palliative care involves bringing together administrative, organisational, clinical and service aspects in order to realise the continuity of care between all actors involved in the care network of patients receiving palliative care. It aims to achieve quality of life and a well-supported dying process for the patient and the family in collaboration with all the caregivers (paid and unpaid)” [76] (p2).

For the purposes of their review, they used Emanuel et al.’s [77] IPC criteria.

The same research team identified 14 empirically tested integrative models [75] that showed the benefits of involving a multidisciplinary palliative care team, namely better symptom control, less caregiver burden, improved continuity and coordination of care, fewer admissions, cost effectiveness and patients dying in their preferred place. On this basis, they proposed a generic framework for integrated palliative care in cancer and chronic disease. Interestingly, however, the framework they propose pays no attention to engaging primary and community care, or informal caregivers, although these contributors are an integral part of both their own definition of ICP above [76] and Emanuel et al.’s [77] criteria.

Other reviews considered integration of care at particular points of the illness trajectory or for specific illness journeys. For example, Gardiner et al. [78] reviewed effective collaborative partnerships between generalist and specialist palliative care services, identified by good communication between providers, clear delineation of roles and responsibilities, opportunities for shared learning and coordination shown in timely access to specialist services. These same conditions are endorsed, albeit by identifying barriers to integrating primary and specialist cancer care, by Dossett et al. [79]. Gardiner et al. [78] did not, however, find any distinctive model of collaborative working undergirding these partnerships. The same team reviewed the transition from curative care to palliative care [78], and again found multidisciplinary collaboration to be a key requirement for navigating this complex transition. Other studies considered integration of respiratory and palliative care [80] and oncology and palliative care [81]. Again, communication and multidisciplinary practice were found to be core elements of successful practice.

A variety of triage tools to assist clinicians in monitoring transitions is being developed. El Mokhallati et al. [82] reviewed 10 screening tools, noting the disparity between those that focus primarily on prognosis and functional decline and those that anticipate palliative needs. They found the predictive ability of all tools to be limited. The recently validated Responding to Urgency of Need in Palliative Care (RUN-PC) triage tool is an important step in making triage more transparent and evidence-based [83]. While these tools support clinical judgement, the pre-conditions of collaborative stance and multidisciplinary practice are essential for their effective use.

The published literature overwhelmingly addresses integration issues that involve health services, with little or no attention paid to integration of these services with informal care. Schulz et al. [84], through a review of family caregiving literature, provided some useful insights into the impact health services can have upon family caregivers as illness progresses and service delivery responds to increasing demand. They found that family caregivers, while still a major source of support to the seriously ill patient, are often marginalised by healthcare systems and procedures. Familiar routines of home care are often now supplemented, or replaced, by further medical and hospital appointments or admissions, additional decision-making about treatment and support and new care issues to be managed at home. The family caregiver also becomes a major source of information for a number of new specialist providers. These additional responsibilities require new knowledge and skills that would ideally be provided by the specialised health service partners being brought into the expanded care network. This introduces a further range of responsibilities that most specialist providers do not recognise and for which they are often not equipped. Yet facilitating partnerships between formal and informal caregivers includes formal caregivers being able to identify and assess the capacity of informal caregivers, support them and equip them to engage with the new challenges.

### 4.3. Appropriateness of Different Models of Palliative Care

The research literature tends to report palliative care outcomes in terms of the setting in which care is received, but both within and across those settings, a range of organisational models can be found [73,85]. Comparisons of models based on such studies can only be performed in general descriptive terms because of the different outcome and process measures used in various studies and different health jurisdictions. As reported above, criteria for evaluating the effectiveness of services usually focus on symptom control and the delivery of clinical care: attention to consumer engagement has been minimal. Brereton et al. [85] note that the majority of evaluation studies demonstrate the overall benefit of providing palliative care compared with other forms of care available in that palliative care service’s health system. There is seldom any information about the components of the palliative intervention or the comparators against which benefit is shown. They conclude:

“Irrespective of setting or patient characteristics, models of palliative care appear to show benefits and some models of palliative care may reduce total healthcare costs. However, much more detailed and systematic reporting of components and agreement about outcome measures is essential to understand the key components and successfully replicate effective organisational models” [85], p.781.

While there is no consensus about organisational models, there is agreement that certain quality standards for care should be the goal of care in any setting. The Australian National Consensus Statement on essentials of end-of-life care [86] applies to all end-of-life care programs, although how care should be provided is not prescribed. The NICE guidelines [87] address what are effectively the same quality standards but do this in ways that make implementation clear and provide links to explore further the rationale for each guideline and the evidence supporting it. Impact findings for the guidelines are also provided [88].

Cost-effectiveness is clearly an important consideration in developing palliative care services. Unfortunately, comparisons of the costs of different palliative care models, as Groeneveld et al. [89] show, encounter problems similar to those identified by Brereton et al. [85]. Funding mechanisms are country-specific and often tenuously aligned with overall health policy [89,90]. It can be shown that palliative care is cost-effective, with savings improved by earlier palliative interventions in cases of multi-morbidity and the use of palliative care strategies that reduce or avoid hospitalisation. For example, in Australia, elderly cancer cohorts incur greater costs at the end-of-life, primarily through hospitalization—those who die in residential aged care incur half the costs of those who die in hospital [91]—although it might also be noted that the survey undertaken for this review indicates a lower quality of care for residents of aged care. Groeneveld et al. [89] suggest six ‘desirable features’ for palliative care funding models:

1. Support early access to palliative care.
2. Provide an appropriate mix of palliative and curative services.
3. Provide services in the most appropriate location.
4. Avoid financial hardship for service users and families.
5. Provide stable and predictable funding that allows coherent planning and development of services.
6. Ensure clarity concerning entitlement to services and ways services can be navigated.

Duckett [90] argues that, for these features to be realised, palliative care funding needs to move toward an activity-based funding model with agreed classification.

It is also notable that these features point toward the integration of specialist and generalist services. As noted in our review, the most recent literature shows a move from comparing settings toward integrating services across the illness journey. This integration seeks to extend the effectiveness and reach of palliative care by better connecting generalist and specialist palliative care in ways that are appropriate to each person’s illness journey. A key component of integration, Luckett et al. [73] suggested, was case management, which is the service element most common to models of care that are effective. It cannot, however, be assumed that providing case management will of itself achieve integration: case management is usually present as part of quite complex and diverse interventions. But it does appear that case management allows consumers to make the best use of the resources available to them.

#### 4.3.1. Involving the Community: Public Health Approaches to End-of-Life Care

It is clear from the evidence concerning end-of-life need that any comprehensive model of care must take very seriously the intersection between health service provision and social network support. Yet, while integrated palliative care guidelines acknowledge the need to involve communities and unpaid caregivers, in practice most service models continue to be designed within the boundaries of the health system.

Over the past decade in particular, palliative care service guidelines have increasingly sought to take consumer experience into account. This has been attempted, for the most part, by collecting data from consumers on their experience, or involving a representative consumer in a reference group, then proceeding to design a health service response to the consumer needs that are of central interest to palliative care practitioners. Public health approaches have a different strategy that focuses less upon identifying patient and family perspectives to which services should develop a response, more upon building systems of care that allow active participation of people and their social networks in the care provided at end-of-life. That is, public health approaches to palliative care pay attention not only to integrating care within health services but also to connecting informal care—a patient’s existing social networks and local community assets—with the care provided by the formal services. By doing this, a public health approach extends issues such as management, accreditation and governance beyond the health sector alone.

A comprehensive public health approach sees people at the centre of care [92]. Rather than individual care, public health approaches give focus and substance to clinical palliative care’s notion of ‘patient-and-family’ as the unit of care, an approach represented in the circles of care model [93]. End-of-life care systems, to be effective, must not only recognise this ‘patient and social network’ context but ensure that professional care, service delivery and policy enhance the care provided by the person’s social network. The unfortunate reality of most health service programs is that they can actively disrupt rather than support that network [94].

Evidence for the effectiveness of this public health approach to care is accumulating rapidly. The scoping review by Daryll Archibald and colleagues [95] is nearing publication. Another overview of relevant evidence and resources is in the report by Nous “Compassionate communities: implementation guide for community approaches to end-of-life care” [96].

#### 4.3.2. Building Community Capacity to Care

Most public health initiatives in palliative care began with identifying community members interested in end-of-life care and building upon their knowledge and skills to communicate these to family, friends and neighbours. Often, although not always, these people were already connected as volunteers with community palliative care services, and through their wider community engagement were able to connect these services with other community groups. One early program, funded by the Victorian Department of Health through the “Strengthening Palliative Care through Health Promotion 2004–2007” program, is outlined in Salau et al. [97].

Around that time, Australian efforts tended to be initiated by palliative care services through their volunteer programs. However, such a strategy has limited community engagement, largely due to the way services have managed volunteer programs. Volunteers have extended the reach of the services more than developed community capacity [98]. Some services in the UK took a different approach, in effect building community capacity with no expectation of ongoing management. Cronin [99] reported on a 2010 experiment to assist local communities by training free-standing volunteer networks to support the most frail and vulnerable in their midst. Their focus was on improving social connection, but they found that consistently after hours call-outs to GP services or trips to EDs declined significantly in the areas where these trained volunteers operated.

The most systematic development of this finding to date has been the connectors project [100] in Frome, Somerset. The sole GP practice in Frome cares for a population of around 28,000 people. The intervention involved rigorous identification of all those in need, not limited by age or diagnosis, followed by care planning and referral to a community development service for goal setting and social network enhancement. This service, Health Connections Mendip (HCM), was established as part of the project. Participants were selected by ‘clinical impression’. Palliative patients are thus part of the mix, not treated as a separate cohort. The GP clinic works in partnership with Health Connections Mendip, which is operated by community development workers known as health connectors. HCM generates and maintains a comprehensive Mendip Directory, offers one-on-one care planning, and trains and then supports volunteer Community Connectors. Health connectors are the bridge between clinic and community; community connectors are conversation partners/promoters of the program. The research team was able to demonstrate over a three-year period a 14% reduction of unplanned admissions to Emergency Departments, compared with a 28.5% increase in the remainder of Somerset.

The Frome project combined capacity building with social network enhancement, informed by work carried out by the Western Sydney University Care at End-of-Life Research Group. Rather than extend the reach of community palliative care services as earlier capacity-building approaches had carried out, the latter group identified social networks in the community that had already cared for a dying friend or relative and explored the experiences of members in the networks [94,101,102,103,104,105,106,107]. They found that effective networks had at least one person with previous experience of dying (not necessarily at home); comprised family, friends and work colleagues; and were most likely to exist when the dying person or primary carer was already embedded in a community with well-established networks (hence the emphasis on enhancing social networks in subsequent public health interventions). Service providers, while essential, were part of the outer network, together with employers and people who worked in local businesses, schools, clubs and community groups (as illustrated in the circles of care diagram).

Wegleitner et al. [108,109] implemented a social networks enhancement project in Landgren, Austria, a town of 8000 people with a strong reputation for civic action but lacking a specialised palliative care service. As part of a large-scale community research project they used a participatory action method first to investigate, and then enhance, the networks through which elderly people were supported in the final years of their lives. They identified ‘ingredients’ of these webs of caring relationships as a focus on relationships and social systems; creating reflective spaces; strengthening social capital; and addressing inequalities in care. All these ingredients, they noted, needed to be cultivated through a process of co-creation. The social networks created a ‘third social space’ between private households and institutional care, but maintaining this space involved resisting the privatisation of care that splits care between commercial and private provision.

Compassionate communities are the descriptor used to capture much of this community development work [108,110,111]. The concept reflects the priority public health gives to settings: if compassion—recognition of our fundamental human connectedness—characterises our communities, then they should be settings in which living and dying can be healthy. The concept has been operationalised in the Compassionate City Charter (https://www.phpci.org/tools, accessed on 28 September 2021) to which a number of UK cities, and increasingly cities in other places, including Australia, have subscribed.

The Compassion Charter is one strategy for developing civic engagement around end-of-life issues. Sallnow and Paul [112] showed how community engagement could range from services providing information to a community through to active co-design of services with a community. The latter is the stance that best reflects a public health approach. Models for citizen-led approaches to health and care include the Wigan local government project [113] and citizens’ juries for health policy decision-making [114]. Co-design has been shown to be effective in developing programs with vulnerable communities [115,116]. End-of-life care applications have been explored by Chung et al. [117] and McCarron et al. [118].

#### 4.3.3. Integrated Public Health Palliative Care

To build a system of end-of-life care that connects health services with community services, and formal care with informal care, further development of palliative care linkages with primary care and aged care, and with civic programs that mobilise and nurture compassionate care in local communities is needed. Tools and resources to create compassionate communities or, more specifically, to create, support and maintain social networks with capacity to care for each other at the end-of-life, have been developed in a variety of jurisdictions. The principal resources are available through Public Health Palliative Care International (https://www.phpci.org/tools accessed on 28 September, 2021). This site also provides links to projects where particular tools are being implemented.

Evidence is being produced but, as with many community-development projects, larger-scale implementation requires longer time scales to produce sustainable change. The core empirical evidence supporting the compassionate communities approach continues to be the social networks studies outlined above, together with case study collections [109,119]. Specific Australian resources include End-of-life Care at Home [104], Compassionate Communities: An implementation guide for community approaches to end-of-life care [96], Greater Choice for At Home Palliative Care Evaluation [120] and the Healthy End-of-life Project [121]. Examples of Australian projects that can contribute to an Essentials Model of Palliative Care [122] include The South West Compassionate Communities Connector Project [123] and The Australian End-of-life and Bereavement Survey [17,18,42,124]. This body of work, innovative in content, conceptual model and recruitment approach, has challenged the existing bereavement support structure and provision and influenced practice and policy in the UK and Ireland [125].

## 5. Discussion

This review, which has an Australian focus, draws upon national and international literature to gather evidence concerning patient preferences for settings in which to receive palliative care (home, residential care, hospital, hospice or a mix) and to review the usefulness of different models of palliative care.

The findings require us to qualify the objectives of the review, which assume that the locus of care—hospital, home, hospice, residential aged care—could lead to different experiences of care and levels of satisfaction with care. In practice, most patients experience a mix of settings during their illness, but the findings of the review have more to do with qualities and values that will contribute to good end-of-life care in any location, not a choice of setting as such. Of course, specific needs may be best met in particular settings, but preferences are based on needs being met at that stage in the illness journey. The hierarchy of need will vary from person to person, and consumers should be involved in negotiating transitions between different locations, recognising that giving priority to one need (for example, hospital admission to adjust medications) may inevitably reduce the extent to which another need is met (security provided by home care). The finding that good care can be achieved in a variety of locations is encouraging, but issues raised by transitions between settings merit further attention.

Evidence for the benefit of extending a palliative approach to all at end-of-life is strong. There is no compelling evidence that points to a preferred organisational model. End-of-life needs of particular groups incorporate the needs associated with their illness or condition. Thus, end-of-life care should be provided collaboratively, led as much as possible by primary care providers with expertise in a person’s illness experience, enhanced by care from those with end-of-life expertise.

There appears to be overall a preference for receiving care at home, but this does not mean that patients or family members are dissatisfied with care in other contexts. While the literature increasingly supports an illness journey perspective, there are insufficient data concerning patients’ overall illness journeys to draw any conclusions about a preferred mix of sites of care. An illness journey perspective is, however, linked with early palliative care referral, perhaps because palliative care can then perform a case management role through the illness course.

Consumer experience of palliative care is investigated poorly, and consumer contribution to service and policy design is limited and selective. It is important that a strategy for receiving consistent and regular consumer feedback, such as FAMCARE-2 or a Palliative Care Experience survey, be introduced into the sector’s Continuous Quality Improvement processes.

The literature examined here agrees on core relational values that should undergird treatment, and that patients’ and families’ needs around information sharing require further practical attention. These needs appear to be independent of setting and to be more related to consumers’ capacity to engage with the process of care, that is, consumers need guidance on how they can contribute, particularly during transitions in care, not merely information about treatment being provided. The literature calls for a person-centred approach from health services but the lack of clarity surrounding this phrase requires more detailed description of what this might involve.

Current literature is less interested in comparing models of care, more interested in the integration of existing approaches to palliative care. Thus the NICE service delivery guidelines for end-of-life care for adults [87], the most recent and comprehensive available, provide evidence and recommend strategy, but emphasise that translation of the guidelines into particular settings requires professional judgement exercised in consultation with the individual and family or carers. The integrated models being put forward in the past few years tend to focus more upon the potential role of primary care in facilitating integration, although there is reference to the place of informal caregivers and community support in meeting consumer need. In the Australian context this suggests the need for Primary Health Networks (PHNs) to be equipped as hubs that connect community services with aged care and palliative care services, and for GPs to be seen as an integral part of end-of-life care. In terms of costs, it seems clear that any palliative care strategy that reduces hospitalisation is likely to be cost-effective. It also seems imperative that aged care services have a greater capacity for end-of-life care on-site and can thus reduce costly non-beneficial hospital admissions. While inappropriate end-of-life care in hospital settings can be addressed to a certain degree by Advance Care Plans that include refusal of non-beneficial treatment, non-beneficial admissions remain a systemic issue that must be addressed beyond the acute care setting.

Some models for integrating different aspects of health services are available. There are fewer models that consider the integration of health services with other social and community services, and fewer again that explore collaboration and integration of formal services with informal networks of care. Despite their rhetoric about community engagement and involvement of informal caregivers, most current models of IPC fall short because their integration is limited to formal health services and consumers are involved as clients to be consulted rather than partners in the co-design of services. Effective integration must include the multiple systems that provide care for a dying person, their family and immediate social network. This raises some largely unexplored questions about the reciprocal contributions of formal and informal care providers, including the mutual recognition of those contributions through role sharing and referral. Formal service providers, while expressing goodwill toward informal networks, do little to establish, support or maintain them.

Public health approaches to end-of-life care have the potential to enhance the integration of services and provide a comprehensive approach that engages the assets of local communities. They also offer frameworks in which partnerships can be developed with communities that have distinctive end-of-life needs, and thus provide a more inclusive approach to end-of-life care. However, it needs to be noted that quite complex governance issues can be involved in collaborations between formal services and informal networks of care. These issues need to be identified and explored in depth so that risks can be mitigated, and the potential of such collaborations be realised. Clearly such approaches resonate with that of community-controlled health organisations that lead indigenous healthcare in Australia [126]. This is of particular relevance to Western Australia, but considerable further work is needed to develop community-controlled partnerships in end-of-life care [127].

## 6. Limitations

As indicated in the methodology section, this rapid review was intended to identify national or international consensus on patient preferences for settings in which to receive palliative care and the appropriateness of different models of palliative care in order to inform end-of-life care policy and service development for the Western Australian Department of Health. Consensus is based on systematic reviews available in English. These findings were supplemented by studies selected to provide further insights into experiences of care or the process of providing care not available in the evidence provided by systematic reviews. The studies were selected for their relevance to gaps in knowledge. There was no formal attempt to assess quality or risk of bias in the studies found through these supplementary searches, but only reasonably robust studies were included in the review, and a note included in several instances where sample size or specific location could be an issue.

In this article, we have not given specific attention to studies in a rural setting or involving indigenous perspectives, both of which have particular relevance to Western Australian policy priorities. Nor have we included studies of under-served populations that are of particular interest in public health approaches. The lack of access to palliative care by Aboriginal and Torres Strait Islanders, rural and regional people, CALD communities, ‘condition-specific’ groups such as dementia or mental illness, and ‘marginalised groups’ such as homeless, refugees, members of the LGBTIQ+ community, has been acknowledged in many WA Health documents across the years. While these population groups have been the subject of a more recent tailored national review into their needs [128], further efforts are needed to document and incorporate consumer feedback from these groups in prospective frameworks on end-of-life care.

We are aware that we have undertaken substantial interpretation in our thematic organisation of the studies included here but given the breadth of the question and the need to cover consumer characteristics, experiences and existing service model studies, we believe this to be justified.

## 7. Conclusions

Evidence gathered through the review points toward a consensus that an optimal end-of-life care system will integrate formal services and informal networks of care along the illness pathway. The stumbling block for such integration continues to be the gulf established by contemporary policy and funding constraints that distinguish between professional and informal care—the former regulated, the latter recognised for the most part only when under professional surveillance. Several localised models that bridge this gulf by creating conditions under which active collaboration can flourish have been developed but are not easily scaled-up under current forms of healthcare governance. The key common element appears to be enabling local communities to negotiate with and adapt service delivery programs that have been established at a national or regional level. That is, good models of care take account of consumer experience not by incorporating generalised evidence but by co-creating services with local communities. While it is important to gather feedback on the consumer experience of end-of-life services through continuous quality assessment, we should put an end to approaches that study consumer experience in order that professionals can design organisational responses. Consumer experience should be incorporated in policy and service design through co-design that enhances the social network supporting dying people and their carers and puts this at the centre of a comprehensive, integrated care model.
